# Measuring factors affecting implementation of health innovations: a systematic review of structural, organizational, provider, patient, and innovation level measures

**DOI:** 10.1186/1748-5908-8-22

**Published:** 2013-02-17

**Authors:** Stephenie R Chaudoir, Alicia G Dugan, Colin HI Barr

**Affiliations:** 1 Department of Psychology, College of the Holy Cross, 1 College St., Worcester, MA, 01610, USA; 2 Connecticut Institute for Clinical and Translational Science, University of Connecticut, Dowling South, Suite 1030, UConn Health Center, 263 Farmington Ave, MC 6233, Farmington, CT, 06030-6233, USA; 3 Center for Health, Intervention, and Prevention, University of Connecticut, 2006 Hillside Road, Unit 1248, Storrs, CT, 06269, USA

**Keywords:** Implementation, Health innovation, Evidence-based practice, Systematic review, Measure, Questionnaire, Scale

## Abstract

**Background:**

Two of the current methodological barriers to implementation science efforts are the lack of agreement regarding constructs hypothesized to affect implementation success and identifiable measures of these constructs. In order to address these gaps, the main goals of this paper were to identify a multi-level framework that captures the predominant factors that impact implementation outcomes, conduct a systematic review of available measures assessing constructs subsumed within these primary factors, and determine the criterion validity of these measures in the search articles.

**Method:**

We conducted a systematic literature review to identify articles reporting the use or development of measures designed to assess constructs that predict the implementation of evidence-based health innovations. Articles published through 12 August 2012 were identified through MEDLINE, CINAHL, PsycINFO and the journal *Implementation Science*. We then utilized a modified five-factor framework in order to code whether each measure contained items that assess constructs representing structural, organizational, provider, patient, and innovation level factors. Further, we coded the criterion validity of each measure within the search articles obtained.

**Results:**

Our review identified 62 measures. Results indicate that organization, provider, and innovation-level constructs have the greatest number of measures available for use, whereas structural and patient-level constructs have the least. Additionally, relatively few measures demonstrated criterion validity, or reliable association with an implementation outcome (*e.g.*, fidelity).

**Discussion:**

In light of these findings, our discussion centers on strategies that researchers can utilize in order to identify, adapt, and improve extant measures for use in their own implementation research. In total, our literature review and resulting measures compendium increases the capacity of researchers to conceptualize and measure implementation-related constructs in their ongoing and future research.

## Background

Each year, billions of dollars are spent in countries around the world to support the development of evidence-based health innovations [1,2]—interventions, practices, and guidelines designed to improve human health. Yet, only a small fraction of these innovations are ever implemented into practice [3], and efforts to implement these practices can take many years [4]. Thus, new approaches are greatly needed in order to accelerate the rate at which existing and emergent knowledge can be implemented in health-related settings around the world.

As a number of scholars have noted, researchers currently face significant challenges in measuring implementation-related phenomena [5-7]. The implementation of evidence-based health innovations is a complex process. It involves attention to a wide array of multi-level variables related to the innovation itself, the local implementation context, and the behavioral strategies used to implement the innovation [8,9]. In essence, there are many ‘moving parts’ to consider that can ultimately determine whether implementation efforts succeed or fail.

These challenges also stem from heterogeneity across the theories and frameworks that guide implementation research. There is currently no single theory or set of theories that offer testable hypotheses about when and why specific constructs will predict specific outcomes within implementation science [5,10]. What does exist in implementation science, however, are a plethora of frameworks that identify general classes or typologies of factors that are hypothesized to affect implementation outcomes (*i.e.*, impact frameworks [5]). Further, within the available frameworks, there is considerable heterogeneity in the operationalization of constructs of interest and the measures available to assess them. At present, constructs that have been hypothesized to affect implementation outcomes are often poorly defined within studies [11,12]. And, the measures used to assess these constructs are frequently developed without direct connection to substantive theory or guiding frameworks and with minimal analysis of psychometric properties, such as internal reliability and construct validity [12].

In light of these measurement-related challenges, increasing the capacity of researchers to both conceptualize and measure constructs hypothesized to affect implementation outcomes is a critical way to advance the field of implementation science. With these limitations in mind, the main goals of the current paper were threefold. First, we expanded existing multi-level frameworks in order to identify a five-factor framework that organizes the constructs hypothesized to affect implementation outcomes. Second, we conducted a systematic review in order to identify measures available to assess constructs that can conceivably act as causal predictors of implementation outcomes, and coded whether each measure assessed any of the five factors of the aforementioned framework. And third, we ascertained the criterion validity—whether each measure is a reliable predictor of implementation outcomes (*e.g.*, adoption, fidelity)—of these measures identified in the search articles.

### A multi-level framework guiding implementation science research

Historically, there has been great heterogeneity in the focus of implementation science frameworks. Some frameworks examine the impact of a single type of factor, positing that constructs related to the individual provider (*e.g.*, practitioner behavior change: Transtheoretical Model [13,14]) or constructs related to the organization (*e.g.*, organizational climate for implementation: Implementation Effectiveness model [15]) impact implementation outcomes. More recently, however, many frameworks have converged to outline a set of multi-level factors that are hypothesized to impact implementation outcomes [9,16-18]. These frameworks propose that implementation outcomes are a function of multiple types of broad factors that can be hierarchically organized to represent micro-, meso-, and macro-level factors.

What, then, are the multi-level factors hypothesized to affect the successful implementation of evidence-based health innovations? In order to address this question, Durlak and DuPre [19] reviewed meta-analyses and additional quantitative reports examining the predictors of successful implementation from over 500 studies. In contrast, Damschroder *et al.*[20] reviewed 19 existing implementation theories and frameworks in order to identify common constructs that affect successful implementation across a wide variety of settings (*e.g.*, healthcare, mental health services, corporations). Their synthesis yielded a typology (*i.e.*, the Consolidated Framework for Implementation Research [CFIR]) that largely overlaps with Durlak and DuPre’s [19] analysis. Thus, although these researchers utilized different approaches—with one identifying unifying constructs from empirical results [20] and the other identifying unifying constructs from existing conceptual frameworks [19]—both concluded that structural- (*i.e.*, community-level [19]; outer-setting [20]), organizational- (*i.e.*, prevention delivery system organizational capacity [19]; inner setting [20]), provider-, and innovation-level factors predict implementation outcomes [19,20].

The structural-level factor encompasses a number of constructs that represent the outer setting or external structure of the broader sociocultural context or community in which a specific organization is nested [3]. These constructs could represent aspects of the physical environment (*e.g.*, topographical elements that pose barriers to clinic access), political or social climate (*e.g.*, liberal versus conservative), public policies (*e.g.*, presence of state laws that criminalize HIV disclosure), economic climate (*e.g.*, reliance upon and stability of private, state, federal funding), or infrastructure (*e.g.*, local workforce, quality of public transportation surrounding implementation site) [21,22].

The organizational-level factor encompasses a number of constructs that represent aspects of the organization in which an innovation is being implemented. These aspects could include leadership effectiveness, culture or climate (*e.g.*, innovation climate, the extent to which organization values and rewards evidence-based practice or innovation [23]), and employee morale or satisfaction.

The provider-level factor encompasses a number of constructs that represent aspects of the individual provider who implements the innovation with a patient or client. We use ‘provider’ as an omnibus term that refers to anyone who has contact with patients for the purposes of implementing the innovation, including physicians, other clinicians (*e.g.*, psychologists), allied health professionals (*e.g.*, dieticians), or staff (*e.g.*, nurse care managers). These aspects could include attitudes towards evidence-based practice [24] or perceived behavioral control for implementing the innovation [25].

The innovation-level factor encompasses a number of constructs that represent aspects of the innovation that will be implemented. These aspects could include the relative advantage of utilizing an innovation above existing practices [26] and quality of evidence supporting the innovation’s efficacy (Organization Readiness to Change Assessment, or ORCA [27]).

But, where does the patient or client fit in these accounts? The patient-level factor encompasses patient characteristics such as health-relevant beliefs, motivation, and personality traits that can impact implementation outcomes [28]^1^. In efficacy trials that compare health innovations to a standard of care or control condition, patient-level variables are of primary importance both as outcome measures of efficacy (*e.g.*, improved patient health outcomes) and as predictors (*e.g.*, patient health literacy, beliefs about innovation success) of these efficacy outcomes. Patient-level variables such as behavioral risk factors (*e.g.*, alcohol use [29]) and motivation [30,31] often moderate the retention in and efficacy of behavioral risk reduction interventions. Moreover, patients’ distrust of medical practices and endorsement of conspiracy beliefs have been linked to poorer health outcomes and retention in care, especially among African-American patients and other vulnerable populations [32]. However, in implementation trials testing whether and to what degree an innovation has been integrated into a new delivery context, the outcomes of interest are different from those in efficacy trials, typically focusing on provider- or organizational-level variables [33,34].

Despite the fact that they focus on different outcomes, what implementation trials have in common with efficacy trials is that patient-level variables are important to examine as predictors, because they inevitably impact the outcomes of implementation efforts [28,35]. The very conceptualization of some implementation outcomes directly implicates the involvement of patients. For example, fidelity, or ‘the degree to which an intervention was implemented as it was prescribed in the original protocol or as it was intended by the program developers’ [6], necessarily involves and is affected by patient-level factors. Further, as key stakeholders in all implementation efforts, patients are active agents and consumers of healthcare from whom buy-in is necessary. In fact, in community-based participatory research designs, patients are involved directly as partners in the research process [36,37]. Thus, as these findings reiterate, patient-level predictors explain meaningful variance in implementation outcomes, making failure to measure these variables as much a statistical as a conceptual omission.

**Figure 1:**
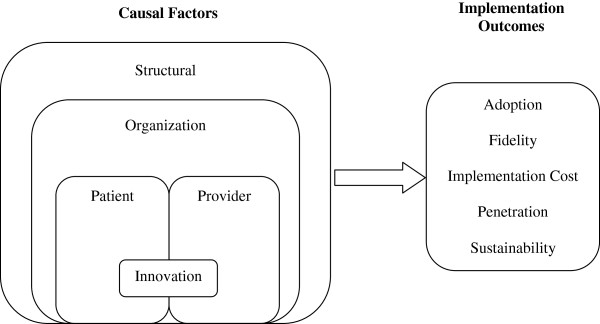
A multi-level framework predicting implementation outcomes.

For the aforementioned reasons, we posit that a comprehensive multi-level framework must include a patient-level factor. Therefore, in the current review, we employed a comprehensive multi-level framework positing five factors representing structural-, organizational-, patient-, provider-, and innovation-levels of analysis. We utilized this five-factor framework as a means of organizing and describing important sets of constructs, as well as the measures that assess these constructs. Figure 1 depicts our current conceptual framework. The left side of the figure depicts causal factors, or the structural-, organizational-, patient-, provider-, and innovation-level constructs that are hypothesized to cause or predict implementation outcomes. These factors represent multiple levels of analysis, from micro-level to macro-level, such that a specific innovation (*e.g.*, evidence-based guideline) is implemented by providers to patients who are nested within an organization (*e.g.*, clinical care settings), which is nested within a broader structural context (*e.g.*, healthcare system, social climate, community norms). The right side of the figure depicts the implementation outcomes—such as adoption, fidelity, implementation cost, penetration, and sustainability [6] — that are affected by the causal factors. Together, these factors illustrate a hypothesized causal effect wherein constructs lead to implementation outcomes.

### Available measures

What measures are currently available to assess constructs within these five factors hypothesized to predict implementation outcomes? The current systematic review seeks to answer this basic question and act as a guide to assist researchers in identifying and evaluating the types of measures that are available to assess structural, organizational, provider, patient, and innovation-level constructs in implementation research.

A number of researchers have also provided reviews of limited portions of this literature [38-41]. For example, French *et al.*[40] focused on the organizational-level by conducting a systematic review to identify measures designed to assess features of the organizational context. They evaluated 30 measures derived from both the healthcare and management/organizational science literatures, and their review found support for the representation of seven primary attributes of organizational context across available measures: learning culture, vision, leadership, knowledge need/capture, acquiring new knowledge, knowledge sharing, and knowledge use. Other systematic reviews and meta-analyses have focused on measures that assess provider-level constructs, such as behavioral intentions to implement evidence-based practices [38] and other research-related variables (*e.g.*, attitudes toward and involvement in research activities) and demographic attributes (*e.g.*, education [41]). Finally, it is important to note that other previous reviews have focused on the conceptualization [6] and evaluation [12] of implementation outcomes, including the psychometric properties of research utilization measures [12].

To date, however, no systematic reviews have examined measures designed to assess constructs representing the five types of factors—structural, organizational, provider, patient, and innovation—hypothesized to predict implementation outcomes. The purpose of the current systematic review is to identify measures available to assess this full range of five factors. In doing so, this review is designed to create a resource that will increase the capacity of and speed with which researchers can identify and incorporate these measures into ongoing research.

## Method

We located article records by searching MEDLINE, PsycINFO, and CINAHL databases and abstracts of articles published in the journal *Implementation Science* through 12 August 2012. There was no restriction on beginning date of this search. (See Additional file 1 for full information about the search process). We searched with combinations of keywords representing three categories: implementation science, health, and measures. Given that the field of implementation science includes terminology contributions from many diverse fields and countries, we utilized thirteen phrases identified as common keywords from Rabin *et al.*’*s* systematic review of the literature [34]: diffusion of innovations, dissemination, effectiveness research, implementation, knowledge to action, knowledge transfer, knowledge translation, research to practice, research utilization, research utilisation, scale up, technology transfer, translational research.

As past research has demonstrated, use of database field codes or query filters is an efficient and effective strategy for identifying high-quality articles for individual clinician use [42,43] and systematic reviews [44]. In essence, these database restrictions can serve to lower the number of ‘false positive’ records identified in the search of the literature, creating more efficiency and accuracy in the search process. In our search, we utilized several such database restrictions in order to identify relevant implementation science-related measures.

In our search of PsycINFO and CINAHL, we used database restrictions that allowed us to search each of the implementation science keywords within the methodology sections of records via PsycINFO (*i.e.*, ‘tests and measures’ field) and CINAHL (*i.e.*, ‘instrumentation’ field). In our hand search of *Implementation Science* we searched for combinations of the keyword ‘health’ and the implementation science keywords in the abstract and title. In our search of *MEDLINE*, we used a database restriction that allowed us to search for combinations of the keyword ‘health,’ the implementation science keywords, and the keywords ‘measure,’ ‘questionnaire,’ ‘scale,’ ‘survey,’ or ‘tool’ within the abstract and title of records listed as ‘validation studies.’ There were no other restrictions based on study characteristics, language, or publication status in our search for article records.

### Screening study records and identifying measures

Article record titles and abstracts were then screened and retained for further review if they met two inclusion criteria: written in English, and validated or utilized at least one measure designed to quantitatively assess a construct hypothesized to predict an implementation science related outcome (*e.g.*, fidelity, exposure [6,34]). Subsequently, retained full-text articles were then reviewed and vetted further based on the same two inclusion criteria utilized during screening of the article records. The remaining full-text articles were then reviewed in order to extract all measures utilized to assess constructs hypothesized to predict an implementation science related outcome. Whenever a measure was identified from an article that was not the original validation article, we used three methods to obtain full information: ancestry search of the references section of article in which the measure was identified, additional database and Internet searches, and directly contacting corresponding authors via email.

### Measure and criterion validity coding

We then coded each of the extracted measures to determine whether it included items assessing each of the five factors—structural, organizational, provider, patient, and innovation—based on our operational definitions noted above. Items were coded as structural-level factors if they assess constructs that represent the structure of the broader sociocultural context or community in which a specific organization is nested. For example, the Organizational Readiness for Change scale [45] assesses several features of structural context in which drug treatment centers exist, including facility structure (independent versus part of a parent organization) and characteristics of the service area (rural, suburban, or urban). Items were coded as organizational level factors if they assess constructs that represent the organization in which an innovation is being implemented. For example, the ORCA [27] assesses numerous organizational constructs including culture (*e.g.*, ‘senior leadership in your organization reward clinical innovation and creativity to improve patient care’) and leadership (*e.g.*, ‘senior leadership in your organization provide effective management for continuous improvement of patient care’). Items were coded as provider-level factors if they assess constructs that represent aspects of the individual provider who implements the innovation. For example, the Evidence-Based Practice Attitudes Scale [24] assesses providers’ general attitudes towards implementing evidence-based innovations (*e.g.*, ‘I am willing to use new and different types of therapy/interventions developed by researchers’) whereas the Big 5 personality questionnaire assesses general personality traits such as neuroticism and agreeableness [46]. Items were coded as patient-level factors if they assess constructs that represent aspects of the individual patients who will receive the innovation directly or indirectly. These aspects could include patient characteristics such as ethnicity or socioeconomic status (Barriers and Facilitators Assessment Instrument [47]), and patient needs (*e.g.*, ‘the proposed practice changes or guideline implementation take into consideration the needs and preferences of patients’; ORCA [27]). Finally, items were coded as innovation-level factors if they assess constructs that represent aspects of the innovation that is being implemented. These aspects could include the relative advantage of an innovation above existing practices [26] and quality of evidence supporting the innovation’s efficacy (ORCA [27]).

The coding process was item-focused rather than construct-focused, meaning that each item was evaluated individually and coded as representing a construct reflecting a structural, organizational, individual provider, individual patient, or innovation-level factor. In order for a measure to be coded as representing one of the five factors, it had to include two or more items assessing a construct subsumed within the higher-order factor. We chose an item-focused coding approach because there is considerable heterogeneity across disciplines and across researchers regarding psychometric criteria for scale development the procedures by which constructs are operationalized.

It is also important to note that we coded items based on the subject or content of the item rather than based on the viewpoint of the respondent who completed the item. For example, a measure could include items in order to assess the general culture of a clinical care organization from two different perspectives—the perspective of the individual provider, or from the perspective of administrators. Though these two perspectives might be construed to represent both provider and organizational-level factors, in our review, both were coded as organizational factors because the subject of the items’ assessment is the organization (*i.e.*, its culture) regardless of who is providing the assessment.

Measures were excluded because items did not assess any of the five factors (*e.g.*, they instead measured an implementation outcome such as fidelity [48]), were utilized only in articles examining a non-health-related innovation (*e.g.*, end-user computing systems [49]), were unpublished or unobtainable (*e.g.*, full measure available only in an unpublished manuscript that could not be obtained from corresponding author [50]), provided insufficient information for review (*e.g.*, multiple example items were not provided in original source article, nor was measure available from corresponding author [51]), were redundant with newer versions of a measure (*e.g.*, Typology Questionnaire redundant with Competing Values Framework [52]), or were only published in a foreign language (*e.g.*, physician intention measure [53]).

In addition, we coded the implementation outcome present in each search article that utilized one of the retained measures in order to determine the relative predictive utility, or criterion validity, of each of these measures [54]. In essence, we wanted to determine whether each measure was reliably associated with one or more implementation outcomes assessed in the articles included in our review. In order to do so, two coders reviewed all search articles and identified which of five possible implementation outcomes was assessed based on the typology provided by Proctor *et al.*[6]^2^: adoption, or the ‘intention, initial decision, or action to try or employ an innovation or evidence-based practice’; fidelity, or ‘the degree to which an intervention was implemented as it was prescribed in the original protocol or as it was intended by the program developers’; implementation cost, or ‘the cost impact of an implementation effort’; penetration, or ‘the integration of a practice within a service setting and its subsystems’; sustainability, ‘the extent to which a newly implemented treatment is maintained or institutionalized within a service setting’s ongoing, stable operations’; or no implementation outcomes. In addition, for those articles that assessed an implementation outcome, we also coded whether each included measure was demonstrated to be a statistically significant predictor of the implementation outcome. Together, these codes indicate the extent to which each measure has demonstrated criterion validity in relation to one or more implementation outcomes.

### Reliability

Together, the first and third authors (SC and CB) independently screened study records, reviewed full-text articles, identified measures within articles, coded measures, and assessed criterion validity. At each of these five stages of coding, inter-rater reliability was assessed by having each rater independently code a random sample representing 25% of the full items [55]. Coding discrepancies were resolved through discussion and consultation with the second author (AD).

## Results

### Literature search results

**Figure 2:**
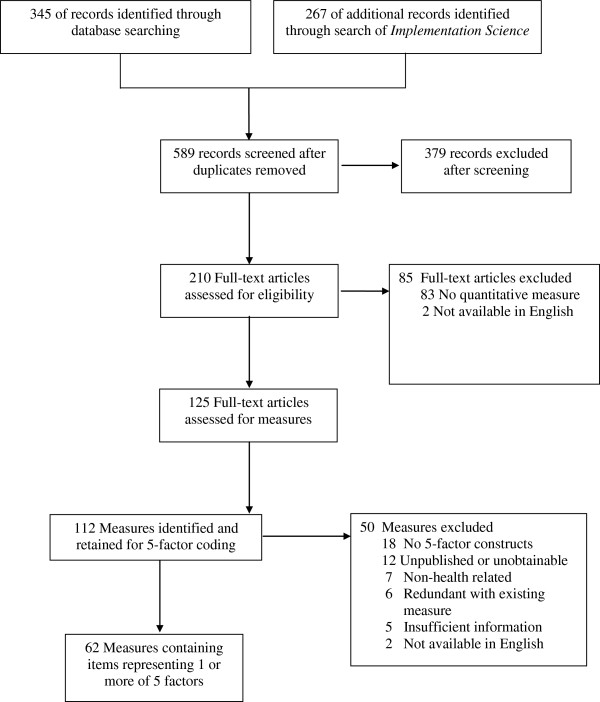
Systematic literature review process.

As depicted in Figure 2, these search strategies yielded 589 unique peer-reviewed journal article records. Of those, 210 full-text articles were reviewed and vetted further, yielding a total of 125 full-text articles from which measures were extracted. A total of 112 measures were extracted from these retained articles. Across each of the stages of coding, inter-rater reliability was relatively high, ranging from 87 to 100% agreement.

Our screening yielded a total of 62 measures. Table 1 provides the full list of measures we obtained. (See Additional file 2 for a list of the names and citations of excluded measures.) For each measure, we provide information about its name and original validation source, whether it includes items that assess each of the five factors, information about the constructs measured, and the implementation context(s) in which the scale was used: healthcare (*e.g.*, nursing utilization of evidence-based practice, guideline implementation), workplace, education, or mental health/substance abuse settings. In addition, we list information about the criterion validity [54] of each measure by examining the original validation source and each search article that utilized the scale, the type of implementation outcome that was assessed in each article, and whether there was evidence that the measure was statistically associated with the implementation outcome assessed. It is important to note that we utilized only the 125 articles eligible for final review and the original validation article (if not located within our search) in order to populate information for the criterion validity and implementation context. Thus, this information represents only information available through these 125 articles and not from an exhaustive search of each measure within the available empirical literature.

**Table 1:** **Coded measures (*N*= 62)**

**Scale name and original source**	**Structural (S)**	**Organizational (O)**	**Individual: Provider (PR)**	**Individual: Patient (PA)**	**Innovation (I)**	**Construct information**	**Search article/s**	**Criterion validity**	**Implementation context**
Alberta Context Tool (ACT) [86]		X				O: culture, leadership, evaluation, social capital, informal interactions, formal interactions, structural and electronic resources, organizational slack	[86]	Adoption*	Healthcare
							[87]	Adoption*	
							[88]	None	
							[89]	None	
							[90]	None	
Appraisal of Guidelines, Research, and Evaluation in Europe (AGREE) scale [91]					X	I: Scope and purpose, stakeholder involvement, rigor of development, clarity and presentation, applicability, editorial independence	[91]	None	EBP government support organizations
							[92]	None	
Attitudes, Perceived Demand, and Perceived Support (ARTAS) [93]	X	X		X	X	S: Funding and policy support	[93]	Adoption*	Healthcare
						O: Management support			
						PA: Patient benefit			
						I: Adaptability and feasibility			
Barriers and Facilitators Assessment Instrument [47]	X	X	X	X	X	S: Social, political, societal context	[47]	Adoption*	Healthcare
							[94]	None	
						O: Organizational context			
						PR: Care provider characteristics			
						PA: Patient characteristics			
						I: Innovation characteristics			
Barriers to Implementation of Behavioral Therapy (BIBT) [95]		X	X	X		O: Institutional constraints, insufficient collegial support	[95]	None	Mental Health/ Substance Abuse
							[96]	None	
						PR: Philosophical opposition to evidence-based practice	[97]	None	
						PA: Client dissatisfaction			
Barriers to Research Utilization Scale (BARRIERS) [56]		X	X		X	O: Setting barriers and limitations	[56]	None	Healthcare
						PR: Research skills, values, and awareness of EBP	[77]	Adoption*	
						I: Quality and presentation of research	[98]	None	
							[99]	None	
							[100]	None	
							[101]	None	
							[102]	None	
							[103]	None	
							[78]	Adoption	
							[79]	None	
							[80]	Adoption*	
							[104]	None	
							[105]	None	
Big 5 Personality (*e.g.*, NEO-FFI) [46]			X			PR: Personality attributes (openness, conscientiousness, extraversion, agreeableness, neuroticism)	[46]	None	Education
							[66]	Fidelity*	
California Critical Thinking Dispositions Inventory [106]			X			PR: Inquisitiveness, systematicity, analyticity, truth-seeking, open-mindedness, self-confidence, maturity	[106]	None	Healthcare
							[107]	Adoption*	
							[108]	None	
Clinical Practice Guidelines Implementation Instrument [64]		X			X	O: Context features	[64]	None	Healthcare
						I: Evidence			
Community-Level Predictors [109]	X					S: Poverty and population	[109]	Adoption*	Healthcare
Competing Values Framework [62] Adapted from [110,111]		X				O: Organizational culture (hierarchical, entrepreneurial, team, and rational)	[62]	None	Healthcare
Context Assessment Index [112]		X				O: Collaborative practice, evidence-informed practice, respect for persons, practice boundaries, evaluation	[112]	None	Healthcare
Coping Style: Supervisory Working Alliance Inventory [113]			X			PR: Coping style	[113]	None	Education
							[66]	Fidelity*	
Decision-Maker Information Needs and Preferences Survey [114]	X	X	X		X	S: Financial resources, impact of regulations and legislation	[114]	None	Healthcare
						O: Support for EBP			
						PR: Preferences for EBP information			
						I: Quality, relevance of EBP			
Dimensions of the Learning Organization Questionnaire [115]		X				O: Continuous learning, inquiry and dialogue, collaboration and team learning, systems to capture learning, empower people, connect the organization, provide strategic leadership for learning, financial performance, knowledge performance	[115]	None	Healthcare
							[116]	Adoption*	
Edmonton Research Orientation Survey (EROS) [58]			X			PR: Valuing research, research involvement, being at the leading edge	[58]	None	Healthcare
							[79]	None	
							[104]	None	
Electronic Health Record Nurse Satisfaction Survey (EHRNS) [117]					X	I: Satisfaction with innovation	[117]	None	Healthcare
EPC [52,118]			X			PR: Cognitive response style	[52]	None	Healthcare
							[118]	Adoption*	
							[119]	None	
Evidence Based Practice Attitude Scale [24]			X			PR: Intuitive appeal of EBP, openness to new practices	[24]	None	Mental Health/ Substance Abuse
							[120]	Adoption*	
							[121]	None	
							[122]	Adoption	
							[123]	None ^‡^	
Evidence-Based Practice Beliefs Scale [57]			X			PR: Attitudes about EBP	[57]	Adoption*	Healthcare
							[124]	None	
							[122]	None	
							[125]	None	
Evidence-Based Practice Questionnaire [126]			X			PR: EBP attitudes, knowledge, and skills	[126]	Adoption*	Healthcare
							[80]	Adoption*	
							[77]	Adoption*	
Facilitators Scale [105]		X	X		X	O: Support for research	[105]	None	Healthcare
						PR: Education	[98]	None	
						I: Improving utility of research	[101]	None	
Four As Research Utilization Survey [127]		X				O: Organizational barriers to access, assess, adapt, and apply EBP	[127]	Adoption	Mental Health/ Substance Abuse
General Practitioners’ Perceptions of the Route of Evidence-Based Medicine [128]		X	X		X	O: Organization support for EBP	[128]	None	Healthcare
						PR: Attitudes toward EBP	[129]	None	
						I: Quality of evidence			
Group Cohesion Scale [130]		X				O: Perceived group attractiveness and cohesiveness	[130]	None	Healthcare
							[131]	None	
GuideLine Implementability Appraisal (GLIA) [132]					X	I: Implementability	[132]	None	Healthcare
							[133]	None	
Healthy Heart Kit Questionnaire [26]		X	X		X	O: Type of practice	[26]	Adoption*	Healthcare
						PR: Perceived confidence and control			
						I: Relative advantage, compatibility, complexity, trialability, observability			
Information System Evaluation Tool [134]					X	I: Usability and usefulness of innovation	[134]	None	Healthcare
Intention to Leave Scale [135]		X	X			O: Work rewards, people at work, work load	[135]	None	Healthcare
						PR: Intention to leave nursing profession	[131]	None	
Job Satisfaction [136]		X	X			O: Communication and decision-making	[136]	None	Healthcare
						PR: Pay, fringe benefits, and promotion, close friends at work	[131]	None	
Knowledge, Attitudes, and Expectations of Web-Assisted Tobacco Interventions [137]					X	I: Knowledge, expectations, actions, networking, information seeking related to innovation	[137]	None	Healthcare
Knowledge Transfer Inventory (Personal Knowledge Transfer subscale) [138]			X			PR: Knowledge acquisition and sharing	[138]	None	Healthcare
							[139]	None	
Knowledge Transfer and Exchange Correlates [140]	X	X	X			S: Policymakers use of EBP, and funding support	[140]	Adoption	Healthcare
						O: Communication and decision-making			
						PR: Research skills, and research activities			
Leader Member Exchange Scale [141]		X				O: Leadership style, work environment	[141]	None	Healthcare
							[139]	None	
Nurses Retention Index [142]			X			PR: Intention to stay in nursing profession	[142]	None	Healthcare
							[131]	None	
Nursing Research Utilization Survey [143]			X			PR: Attitudes towards nursing research	[143]	Adoption*	Healthcare
Nursing Work Index [144]		X				O: hospital characteristics	[144]	None	Healthcare
							[83]	None	
							[145]	None	
Organization Readiness to Change Assessment (ORCA) [27]		X		X	X	O: culture, leadership, measurement, readiness for change, resources, characteristics, role	[27]	None	Healthcare
						PA: Evidence: Patient preferences	[146]	Adoption*	
						I: Evidence: Disagreement, evidence, clinical experience			
Organizational Culture and Readiness for System-Wide Implementation of EBP (OCRSIEP) [147]		X				O: organizational culture, readiness for system-wide integration of EBP	[147]	None	Healthcare
							[148]	Adoption*	
							[131]	None	
Organizational Culture Survey [60,149]		X				O: Constructive Culture (motivation, individualism, support), Passive Defensive Culture (consensus, conformity, subservience)	[149]	None	Education
							[60]	None	
							[66]	Fidelity*	Healthcare
Organizational Learning Survey (OLS) [150]		X				O: Clarity of purpose, leadership, experimentation and rewards, transfer of knowledge, teamwork	[150]	None	Workplace
							[145]	None	
Organizational Readiness for Change [45]		X	X			O: Institutional resources, Organizational climate, motivational readiness	[45]	None	Mental Health/ Substance Abuse
							[78]	Adoption	
						PR: Staff personality attributes	[151]	Adoption*	
Organizational Social Context† [152]		X	X			O: Climate, culture	[152]	None	Mental Health/ Substance Abuse
						PR: Work attitudes	[121]	None	
Organizational/Psychological Climate [60,153]			X			PR: Job satisfaction, depersonalization, emotional exhaustion, role conflict	[60]	None	Education
							[153]	None	
							[66]	Fidelity*	Mental Health/ Substance Abuse
							[151]	Adoption*	
Ottawa Acceptability of Decision Rules Instrument (OADRI) [154]					X	I: Acceptability of clinical practice guidelines	[154]	Adoption*	Healthcare
Perceived Importance of Dissemination Activities [155]			X			PR: Perceived importance of dissemination activities	[155]	None	University Health Researchers
Pre-Implementation Expectancies [59]		X	X		X	O: Teacher morale, leadership encouragement	[59]	Adoption*	Education
								Fidelity*	
						PR: Enthusiasm for Implementation, preparedness to implement, implementation self-efficacy	[66]	Fidelity*	
						I: Compatibility, beliefs about the program			
Quality Improvement Implementation Survey [156]		X				O: Culture, leadership, information and analysis, strategic planning quality, human resource utilization, quality management, quality results, customer satisfaction	[156]	None	Healthcare
							[157]	None	
Rational Experiential Inventory [158]			X			PR: Rational and experiential thinking styles	[158]	None	Healthcare
							[159]	Adoption*	
Research Conduct and Research Utilization by Nurses Questionnaire [160]			X			PR: Knowledge base for research, attitude towards research utilization	[160]	None	Healthcare
Research Knowledge, Attitudes and Practices of Research Survey [161]			X			PR: Research knowledge, attitudes, practice	[161]	None	Healthcare
							[79]	None	
							[104]	Adoption*	
Research Utilization Questionnaire (RUQ) [162]			X			PR: perceived difficulty of research utilization activities, attitudes regarding utilization	[162]	None	Healthcare
Research Utilization Questionnaire (RUQ) [65]		X	X			O: availability and support	[65]	None	Healthcare
						PR: attitude	[163]	None	
							[108]	Adoption*	
							[164]	None	
San Francisco Treatment Research Center Course Evaluation [165]		X	X			O: Organizational barriers to adopting EBP, agency	[165]	None	Mental Health/ Substance Abuse
							[166]	None	
						Management, strategies to support EBP			
						PR: Stage of change for using EBPs, attitudes regarding EBP,			
						past experience with EBP			
Team Check-Up Tool [167]		X				O: Leadership, shared decision-making, shared vision	[167]	None	Healthcare
							[83]	Adoption* ^‡^	
Team Climate Inventory [168]		X				O: Shared vision, shared decision-making, support, information sharing	[168]	Adoption*	Healthcare
							[123]	None ^‡^	
Team Functioning Survey [169]		X				O: Team skill, support, and work environment	[169]	Adoption*	Healthcare
							[83]	None	
Team Organization and Support Conditions Questionnaire [61]		X				O: organizational support, team organization, external change agent support	[61]	None	Healthcare
Theoretical-Domains Framework [170]		X	X	X		O: Management support, organizational support and resources	[170]	None	Healthcare
						PR: Perceived knowledge, skills, and abilities, motivation			
						PA: patient interest in treatment			
Theory of Planned Behavior Constructs (*i.e.*, attitudes, norms, perceived behavioral control) [25]			X			PR: Attitudes, norms, perceived behavioral control	[25]	None	Healthcare
							[171]	Adoption*	
							[38]	Adoption*	
							[172]	Adoption	
							[173]	Adoption*	
Therapist Characteristics and Barriers to Implementation [174]		X	X			O: clinic type, location, organizational support	[174]	None	Mental Health/ Substance Abuse
						PR: Perceived skills and ability, counseling discipline, level of education, workload, motivation			
Worksite Health Promotion Capacity Instrument (WHPCI) - Health Promotion Willingness subscale [67]		X				O: Health promotion willingness	[67]	Adoption*	Workplace

*Notes*. EBP = evidence based practice. *Measure was a statistically significant predictor of the implementation outcome noted. † This scale includes the organizational culture and organizational climate scales also developed by the same author (Glisson & James, 2002). ^‡^Measure used to predict a patient health outcome.

### Factors assessed

Of the 62 measures we obtained, most (42; 67.7%) assessed only one type of factor. Only one measure—the Barriers and Facilitators Assessment Instrument [47]—included items designed to assess each of the five factors examined in our review.

Of the five factors coded in our review, individual provider and organizational factors were the constructs most frequently assessed by these measures. Thirty-five (56.5%) measures assessed provider-level constructs, such as research-related attitudes and skills [56-58], personality characteristics (*e.g.*, Big 5 Personality [46], and self-efficacy [59]).

Thirty-seven (59.7%) measures assessed organizational-level constructs. Aspects of organizational culture and climate were assessed frequently [45,60] as were measures of organizational support or ‘buy in’ for implementation of the innovation [61-63].

Innovation-level constructs were measured by one quarter of these measures (16; 25.8%). Many of these measures assessed constructs outlined in Roger’s diffusion of innovations theory [18] such as relative advantage, compatibility, complexity, trialability, and observability [26].

Structural-level (5; 8.1%) and patient-level (5; 8.1%) constructs were the least likely to be assessed. For example, the Barriers and Facilitators Assessment Instrument [47] assesses constructs associated with each of the five factors, including structural factors such as the social, political, societal context and patient factors such as patient characteristics. The ORCA [27] assesses patient constructs in terms of the degree to which patient preferences are addressed in the available evidence supporting an innovation.

### Implementation context and criterion validity

Consistent with our search strategies, most (47; 75.8%) measures were developed and/or implemented in healthcare-related settings. Most measures were utilized to examine factors that facilitate or inhibit adoption of evidence-based clinical care guidelines [56,64,65]. However, several measures were utilized to evaluate implementation of health-related innovations in educational (*e.g.*, implementation of a preventive intervention in elementary schools [66]), mental health (technology transfer in substance abuse treatment centers [45]), workplace (*e.g.*, willingness to implement worksite health promotion programs [67]), or other settings.

Surprisingly, almost one-half (30; 48.4%) of the measures located in our search neither assessed criterion validity in their original validation studies nor in the additional articles located in our search. That is, for the majority of these measures, implementation outcomes such as adoption or fidelity were not assessed in combination with the measure in order to determine whether the measure is reliably associated with an implementation outcome. Of the 32 measures for which criterion validity was examined, adoption was the most frequent (29 of 32; 90.1%) implementation outcome examined. Only a small proportion of studies (5 of 32; 15.6%) examined fidelity, and no studies examined implementation cost, penetration, or sustainability. Again, it is important to note that we did not conduct an exhaustive search of each measure to locate all studies that have utilized it in past research, so it is possible that the criterion validity of these measures has, in fact, been assessed in other studies that were not located in our review. In addition, it is important to keep in mind that the criterion validity of recently developed scales may be weak solely because these measures have been evaluated less frequently than more established measures.

## Discussion

Existing gaps in measurement present a formidable barrier to efforts to advance implementation science [68,69]. In the current review, we addressed these existing gaps by identifying a comprehensive, five-factor multi-level framework that builds upon converging evidence from multiple previous frameworks. We then conducted a systematic review in order to identify 62 available measures that can be utilized to assess constructs representing structural-, organizational-, provider-, patient-, and innovation-level factors—factors that are each hypothesized to affect implementation outcomes. Further, we evaluated the criterion validity of each measure in order to determine the degree to which each measure has, indeed, predicted implementation outcomes such as adoption and fidelity. In total, the current review advances understanding of the conceptual factors and observable constructs that impact implementation outcomes. In doing so, it provides as useful tool to aid researchers as they determine which of five types of factors to examine and which measures to utilize in order to assess constructs within each of these factors (see Table 1).

### Available measures

In addition to providing a practical tool to aid in research design, our review highlights several important aspects about the current state of measurement in implementation science. While there is a relative preponderance of measures assessing organizational-, provider-, and innovation-level constructs, there are relatively few measures available to assess structural- and patient-level constructs. Structural-level constructs such as political norms, policies, and relative resources/socioeconomic status can be important macro-level determinants of implementation outcomes [9,19,20]. Why, then, did our search yield so few available measures of structural-level constructs? Structural-level constructs are among the least likely to be assessed because their measurement poses unique methodological challenges for researchers. In order to ensure enough statistical power to test the effect of structural-level constructs on implementation outcomes, researchers must typically utilize exceptionally large samples that are typically difficult to obtain [17]. Alternatively, when structural-level constructs are deemed to be important determinants of implementation outcomes, researchers conducting implementation trials may simply opt to control for these factors in their study designs by stratifying or matching organizations on these characteristics [70] rather than measuring them. Though the influence of some structural-level constructs might be captured through formative evaluation [71], many structural-level constructs such as relative socioeconomic resources and population density can be assessed with standardized population-type measures such as those assessed in national surveys such as the General Social Survey [72].

Though patient-level constructs may be somewhat easier to assess, there is also a relative dearth of measures designed to assess these constructs. Though we might assume that most innovations have been tested for patient feasibility in prior stages of research or formative evaluation [71], this is not always a certainty. Thus, measures that assess the degree to which innovations are appropriate and feasible with the patient population of interest are especially important. Beyond feasibility, other important patient characteristics such as health literacy may also affect implementation, making it more likely that an innovation will be effectively implemented with some types of patients but not others [3]. Measures that assess these and other patient-level constructs will also be useful in strengthening these existing measurement gaps.

In addition to locating those measures outlined in Table 1, the current review also highlights additional strategies that will allow researchers to further expand the available pool of measures. Though measures utilized in research examining non-health related innovations were excluded from the current review, many of these measures (see Additional file 2)—and those identified in other systematic reviews [38,40]—could also be useful to researchers to the extent that they are psychometrically sound and can be adapted to contexts of interest. Further, adaption of psychometrically sound measures from other literatures assessing organizational-level constructs (*e.g.*, culture [73,74]), provider-level constructs (*e.g.*, psychological predictors of behavior change [75,76]), or others could also offer fruitful measurement strategies.

### Criterion validity

Because of its primary relevance in the current review, we evaluated the criterion validity of the identified measures. Our review concluded that for a vast majority of measures, criterion validity has either not been examined or has not been supported. For example, although the BARRIERS scale was the most frequently utilized measure of those included in our review (*i.e.*, utilized in 12 articles), only three of those articles utilized the measure to predict an implementation outcome (*i.e.*, adoption [77-80]). Instead, this measure was used to describe the setting as either amenable or not amenable to implementation, though no implementation activity was assessed by the measure itself. Thus, there is a preponderance of scales that currently serve descriptive purposes only. Though descriptive information obtained through these measures is useful for elicitation efforts, these measures might also provide important information if they are used as predictors of implementation outcomes.

The lack of criterion validity associated with the majority of the measures identified in the current review contributes to growing evidence regarding the weak psychometric properties of many available implementation science measures. As Squires *et al.*[12] recently discussed in their review of measures assessing research utilization, a large majority of these existing measures demonstrate weak psychometric properties. Basic psychometric properties—reliability (*e.g.*, internal reliability, test-retest reliability) and validity (*e.g.*, construct validity, criterion validity)—of any measure should always be evaluated prior to including the measure in research [54]. This is especially true in the area of implementation science measurement, given that it is a relatively new area of study and newly developed measures may have had limited use. Thus, our review highlights the need for continued development and refinement of psychometrically sound measures for use in implementation science settings.

Our examination of implementation outcomes also provides us with a unique opportunity to identify trends in the type and frequency of implementation outcomes used within this sample of the literature. Measures identified in the current review have been predominantly developed and tested in relation to implementation outcomes that occur early in the implementation process (*i.e.*, adoption, fidelity) rather than those outcomes that occur later in the implementation process (*i.e.*, sustainability [81,82]). Presumably, our use of broad search terms such as ‘implementation’ or ‘translational research’ would have identified measures that have been utilized by researchers examining both early (*e.g.*, adoption, fidelity) and later (*e.g.*, sustainability) stages of implementation. To that extent, our findings mirror the progression of the field of implementation science as a whole, with early theorizing and research focusing predominantly on the initial adoption of an innovation and more recent investigations giving greater attention to the long-term sustainability of the innovation. Future research will benefit by examining the degree to which the measures identified in our search (and the constructs they assess) affect later-stage outcomes such as penetration and sustainability in implementation trials or affect patient health and implementation outcomes simultaneously in hybrid effectiveness-implementation designs [33]. In addition, though we did not include patient health outcomes in our criterion validity coding scheme, we did come across a number of articles that included these outcomes (*e.g.*, number of bloodstream infections [83]) alone or in combination with other implementation outcomes. The use of these outcome measures underscores the notion that patient-level variables continue to be relevant in implementation trials as they are in efficacy trials.

### Limitations and future directions

The current review advances implementation science measurement by identifying a comprehensive, multi-level conceptual framework that articulates factors that predict implementation outcomes and provides a systematic review of quantitative measures available to assess constructs representing these factors. It is important to note, however, that the specific search strategies adopted in this systematic review affect the types of articles located and the conclusions that can be drawn from them in important ways.

Our use of multiple databases (*i.e.*, MEDLINE, CINAHL, PsycINFO) which span multiple disciplines (*e.g.*, medicine, public health, nursing, psychology) and search of the *Implementation Science* journal provides a broad cross-section of the empirical literature examining implementation of health-related innovations. Despite this broad search, it is possible that additional relevant literature and measures could be identified in future reviews through the use of other databases such as ERIC or Business Source Complete, which may catalogue additional health- and non-health implementation research from other disciplines such as education and business, respectively. Indeed, a recent review of a wide array of organizational literatures yielded 30 measures of organizational-level constructs [40], only 13% of which overlapped with the current review. Thus, additional reviews that draw on non-health-related literatures will help to identify additional potentially relevant measures.

Further, the specific keywords and database restrictions used to search for these keywords also impact the range of articles identified in the current review. For example, our use of the keyword ‘health’ was designed to provide a general cross-section of measures that would be relevant to researchers examining health-related innovations, broadly construed. Its use may have omitted articles that utilized only a specific health discipline keyword (*e.g.*, cancer, diabetes, HIV/AIDS) but not ‘health’ as a keyword in the title or abstract. Similarly, our measure-related keywords or thirteen expert-identified implementation science keywords [34] may have captured a majority, but not a complete, range of implementation science articles. We did not account for truncation or spelling variants in our search and, in two databases, we limited our search to two instrument-related fields that could have also resulted in missing potentially relevant studies and measures. Additional systematic reviews that utilize expanded sets of keywords (*e.g.*, use of keywords as medical subject headings [MeSH]) may yield additional measures to complement those identified in the current review. Identification of qualitative measures would also further complement the current review.

Finally, as noted earlier, assessment of criterion validity was based only on articles that were identified in the current search. Thus, because separate systematic searches were not conducted on each of the 62 individual measures, our assessment of criterion validity is based on a sampling of the literature available from the search strategy adopted herein. As a consequence, further research would be required in order to ensure an exhaustive assessment of criterion validity for some or all of the identified measures.

## Conclusion

As the nexus between research and practice, the field of implementation science plays a critical role in advancing human health. Though it has made tremendous strides in its relatively short life span, the field also continues to face formidable challenges in refining the conceptualization and measurement of the factors that affect implementation success [84]. The current research addresses these challenges by outlining a comprehensive conceptual framework and the associated measures available for use by implementation researchers. By helping researchers gain greater clarity regarding the conceptual factors and measured variables that impact implementation success, the current review may also contribute towards future efforts to translate these frameworks into theories. As has been demonstrated in nearly every domain of health, theory-based research—in which researchers derive testable hypotheses from theories or frameworks that provide a system of predictable relationships between constructs—has stimulated many of the greatest advances in effective disease prevention and health promotion efforts [85]. So, too, must implementation science translate existing frameworks into theories that can gain greater specificity in predicting the interrelations among the factors that impact implementation success and, ultimately, improve human health.

## Endnotes

^1^It is important to note that we do not consider patient perceptions of the innovation as patient-level factors [28] because the object of focus is the innovation rather than the patient per se. Thus, patient perceptions of the innovation—similar to provider perceptions of the innovation—are considered to be innovation-level factors.

^2^Given that Proctor’s [6] conceptualization of the constructs of acceptability, appropriateness, and feasibility are redundant with our conceptualization of innovation-level factors, we omitted these three constructs from our coding of implementation outcomes.

## Competing interests

The authors declare that they have no competing interests.

## Authors’ contributions

SC and AD designed the study, and all three authors contributed to the development of search strategies. SC acted as the lead writer, with edits and revisions from AD and CB. CB screened all search articles and SC and CB coded all measures in order to determine their assessment of constructs representing the five factors, implementation outcomes assessed, and criterion validity. All authors provided critical commentary on the manuscript and approved the final version.

## Supplementary Material

Additional file 1 Literature search strategies. Click here for file

Additional file 2 Excluded scales and reason for exclusion. Click here for file
