# Supplementary material for: Measuring factors affecting implementation of health innovations: a systematic review of structural, organizational, provider, patient, and innovation level measures
Source: Implement Sci. 2013 Feb 17;8:22. doi: 10.1186/1748-5908-8-22 (PMC3598720; doi:10.1186/1748-5908-8-22)
Supplement: Additional file 1 — Literature search strategies. [file 1748-5908-8-22-S1.doc]

**Additional File 1. Literature search strategies**

**EBSCO HOST: PsycINFO < *all* to August 12, 2012> Search Strategy:**

1. diffusion of innovations in TM Test & Measures
2. dissemination in TM Test & Measures
3. effectiveness research in TM Test & Measures
4. implementation in TM Test & Measures
5. knowledge to action in TM Test & Measures
6. knowledge transfer in TM Test & Measures
7. knowledge translation in TM Test & Measures
8. research to practice in TM Test & Measures
9. research utilization in TM Test & Measures
10. research utilisation in TM Test & Measures
11. scale up in TM Test & Measures
12. technology transfer in TM Test & Measures
13. translational research in TM Test & Measures

**EBSCO HOST: CINAHL with full text < *all* to August 12, 2012> Search Strategy:**

1. diffusion of innovations in IN Instrumentation
2. dissemination in IN Instrumentation
3. effectiveness research in IN Instrumentation
4. implementation in IN Instrumentation
5. knowledge to action in IN Instrumentation
6. knowledge transfer in IN Instrumentation
7. knowledge translation in IN Instrumentation
8. research to practice in IN Instrumentation
9. research utilization in IN Instrumentation
10. research utilisation in IN Instrumentation
11. scale up in IN Instrumentation
12. technology transfer in IN Instrumentation
13. translational research in IN Instrumentation

**Web of Science: MEDLINE < *all* to August 11, 2012> Search Strategy:**

1. “diffusion of innovations” in Topic, limited to validation studies only
2. dissemination in Topic, limited to validation studies only
3. “effectiveness research” in Topic, limited to validation studies only
4. implementation in Topic, limited to validation studies only
5. “knowledge to action” in Topic, limited to validation studies only
6. “knowledge transfer” in Topic, limited to validation studies only
7. “knowledge translation” in Topic, limited to validation studies only
8. “research to practice” in Topic, limited to validation studies only
9. “research utilization” in Topic, limited to validation studies only
10. “research utilisation” in Topic, limited to validation studies only
11. “scale up” in Topic, limited to validation studies only
12. “technology transfer” in Topic, limited to validation studies only
13. “translational research” in Topic, limited to validation studies only

*Results from 14 transferred to Endnote*

1. questionnaire in abstract or title AND health in abstract or title
2. scale in abstract or title AND health in abstract or title
3. measure in abstract or title AND health in abstract or title
4. tool in abstract or title AND health in abstract or title
5. survey in abstract or title AND health in abstract or title

*Note*: Topic field includes Title, Abstract, and Keyword Field.

**Journal *Implementation Science* < *all* to August 11, 2012> Search Strategy:**

1. diffusion of innovations within citation and abstract
2. dissemination within citation and abstract
3. effectiveness research within citation and abstract
4. implementation within citation and abstract
5. knowledge to action within citation and abstract
6. knowledge transfer within citation and abstract
7. knowledge translation within citation and abstract
8. research to practice within citation and abstract
9. research utilization within citation and abstract
10. research utilisation within citation and abstract
11. scale up within citation and abstract
12. technology transfer within citation and abstract
13. translational research within citation and abstract

*Results from steps 1 to 13 transferred to Endnote*

1. diffusion of innovations in abstract or title AND health in abstract or title
2. dissemination in abstract or title AND health in abstract or title
3. effectiveness research in abstract or title AND health in abstract or title
4. implementation in abstract or title AND health in abstract or title
5. knowledge to action in abstract or title AND health in abstract or title
6. knowledge transfer in abstract or title AND health in abstract or title
7. knowledge translation in abstract or title AND health in abstract or title
8. research to practice in abstract or title AND health in abstract or title
9. research utilization in abstract or title AND health in abstract or title
10. research utilisation in abstract or title AND health in abstract or title
11. scale up in abstract or title AND health in abstract or title
12. technology transfer in abstract or title AND health in abstract or title
13. translational research in abstract or title AND health in abstract or title

*Note*: Search steps 1-13 were conducted on the *Implementation Science* website. All records were then transferred to and searched again within *Endnote* in order to replicate the search steps conducted in *PsycInfo* and *CINAHL. Endnote* treats all keywords as though they are surrounded by quotations, serving to limit results to only records possessing the exact phrase listed in the keyword.
