# Supplementary material for: Measuring factors affecting implementation of health innovations: a systematic review of structural, organizational, provider, patient, and innovation level measures
Source: Implement Sci. 2013 Feb 17;8:22. doi: 10.1186/1748-5908-8-22 (PMC3598720; doi:10.1186/1748-5908-8-22)
Supplement: Additional file 2 — Excluded scales and reason for exclusion. [file 1748-5908-8-22-S2.doc]

**Additional File 2. Excluded scales and reason for exclusion.**

| **Reason for Exclusion** | **Scale Names and Citations** |
| --- | --- |
| Did not assess any of 5 Factors (*N* = 18) | Barriers to Treatment Integrity Implementation Survey |
|  | Clinical Strategies Implementation Scale |
|  | Collaborative Research Process Survey |
|  | Implementation Assessment Survey |
|  | Quality of Supported Employment Implementation Scale |
|  | Physician Guidelines Measure |
|  | Process Evaluation Checklist |
|  | Readiness to Change |
|  | Implementation of Treatment Integrity Procedures Scale |
|  | Linderman's Delphi Survey |
|  | Research Utilisation Survey |
|  | Chart Audit Tool |
|  | Clubhouse Research and Evaluation Screening Survey |
|  | Conceptual Research Utilization |
|  | Systems of Care Implementation Survey (SOCIS) |
|  | Level of Institutionalization Survey |
|  | Ducker’s quality improvement questionnaire |
|  | Quality Improvement Questionnaire |
|  |  |
| Redundant with existing measure (*N* = 6) | Typology Questionnaire |
|  | Conduct and Utilization in Nursing Scale (CURN) |
|  | Quality Improvement Implementation Scale |
|  | Sources of knowledge questionnaire |
|  | McKenzie measure of TBP constructs |
|  | Cobban's Composite Questionnaire |
| Unpublished (*N* = 12) | Manual Treatment Attitudes Scale |
|  | Protocol Implementation Scale |
|  | Research Utilization Competence Scale |
|  | Attitudes to Research Scale |
|  | Organizational Support for EBP |
|  | e-Health state of readiness questionnaire |
|  | Conditions of Work Effectiveness Questionnaire-II |
|  | Community Measures |
|  | Modified Practice Attitudes Scale |
|  | Implementation Index |
|  | Coping With Setbacks Work Questionnaire |
|  | Research Factor Questionnaire |
| Non-health related innovation (*N* = 7) | End User Satisfaction |
|  | Implementation Attitudes |
|  | Perceived Ease of Use Survey |
|  | Perceived Usefulness Survey |
|  | Planning and Implementing Phases for Computer Assisted Instruction |
|  | IT Capacities Assessment Tool |
|  | Pretest for Attitudes Toward Computers in Health-Care Assessment Scale |
| Insufficient Information (*N* = 5) | Multi-Dimensional Leader Questionnaire |
|  | Nursing Practice Questionnaire |
|  | Factors Related to Protocol Use Scale |
|  | HERG Questionnaire |
|  | Patient Education Measure |
| Not available in English (*N* = 2) | Physician Intention Scale |
|  | Rogers' Diffusion of Innovation constructs |

**REFERENCES**

1. Perepletchikova F, Hilt LM, Chereji E, Kazdin AE: **Barriers to implementing treatment integrity procedures: Survey of treatment outcome researchers.** *J Consult Clin Psychol* 2009, **77:**212-218.

2. Falloon IRH, Economou M, Palli A, Malm U, Mizuno M, Murakami M: **The clinical strategies implementation scale to measure implementation of treatment in mental health services.** *Psychiatr Serv* 2005, **56:**1584-1590.

3. Blevins D, Farmer M, Edlund C, Sullivan G, Kirchner J: **Collaborative research between clinicians and researchers: A multiple case study of implementation.** *Implem Sci* 2010, **5:**76.

4. Larson EL, Quiros D, Giblin T, Lin S: **Relationship of antimicrobial control policies and hospital and infection control characteristics to antimicrobial resistance rates.** *Am J Crit Care* 2007, **16:**110-120.

5. Bond GR, Campbell K, Evans LJ, Gervey R, Pascaris A, Tice S, Del Bene D, Revell G: **A scale to measure quality of supported employment for persons with severe mental illness.** *J Vocat Rehabil* 2002, **17:**239-250.

6. National Heart Foundation of Australia: **Guidelines for the management of acute coronary syndromes 2006.** *Med J Aust* 2006, **184:**S1-S30.

7. Yamada J, Stevens B, Sidani S, Watt-Watson J, de Silva N: **Content validity of a process evaluation checklist to measure intervention implementation fidelity of the EPIC intervention.** *Worldviews Evid Based Nurs* 2010, **7:**158-164.

8. Forsberg L, Halldin J, Wennberg P: **Psychometric properties and factor structure of the Readiness to Change Questionnaire.** *Alcohol Alcohol* 2003, **38:**276-280.

9. Perepletchikova F, Treat TA, Kazdin AE: **Treatment integrity in psychotherapy research: Analysis of the studies and examination of the associated factors.** *J Consult Clin Psychol* 2007, **75:**829-841.

10. Lindeman CA: **Delphi survey of priorities in clinical nursing research.** *Nurs Res* 1975, **24:**434-441.

11. Estabrooks CA: **Modeling the individual determinants of research utilization.** *West J Nurs Res* 1999, **21:**758-772.

12. Higuchi KS, Edwards N, Danseco E, Davies B, McConnell H: **Development of an evaluation tool for a clinical practice guideline on nursing assessment and device selection for vascular access.** *J Infus Nurs* 2007, **30:**45-54.

13. Macias C, Propst R, Rodican C, Boyd J: **Strategic planning for ICCD clubhouse implementation: development of the Clubhouse Research and Evaluation Screening Survey (CRESS). International Center for Clubhouse Development.** *Mental health services research* 2001, **3:**155-167.

14. Squires JE, Estabrooks CA, Newburn-Cook CV, Gierl M: **Validation of the conceptual research utilization scale: an application of the standards for educational and psychological testing in healthcare.** *BMC Health Serv Res* 2011, **11:**107.

15. Boothroyd RA, Greenbaum PE, Wang W, Kutash K, Friedman RM: **Development of a measure to assess the implementation of children’s systems of care: The Systems of Care Implementation Survey (SOCIS).** *J Behav Health Serv Res* 2011, **38:**288-302.

16. Fortney J, Enderle M, McDougall S, Clothier J, Otero J, Altman L, Curran G: **Implementation outcomes of evidence-based quality improvement for depression in VA community based outpatient clinics.** *Implem Sci* 2012, **7:**30.

17. Duckers M, Wagner C, Vos L, Groenewegen P: **Understanding organisational development, sustainability, and diffusion of innovations within hospitals participating in a multilevel quality collaborative.** *Implem Sci* 2011, **6:**18.

18. Meurer SJ, Rubio D, Counte MA, Burroughs T: **Development of a healthcare quality improvement measurement tool: results of a content validity study.** *Hosp Top* 2002, **80:**7-13.

19. Green L, Gorenflo D, Wyszewianski L: **Validating an instrument for selecting interventions to change physician practice patterns: A Michigan consortium for family practice research study.** *J Fam Pract* 2002, **51:**938-942.

20. Crane J, Pelz D, Horsley JA: *CURN Project Research Utilization Questionnaire.* Ann Arbor, MI: Conduct and Utilization of Research in Nursing Project, School of Nursing. The University of Michigan; 1977.

21. Shortell SM, Obrien JL, Carman JM, Foster RW, Hughes EFX, Boerstler H, Oconnor EJ: **Assessing the impact of continuous quality improvement total quality management: Concept versus implementation.** *Health Serv Res* 1995, **30:**377-401.

22. Estabrooks CA: **Will evidence-based nursing practice make practice perfect?** *Can J Nurs Res* 1998, **30:**15-36.

23. McKenzie J, O'Connor D, Page M, Mortimer D, French S, Walker B, Keating J, Grimshaw J, Michie S, Francis J, Green S: **Improving the care for people with acute low-back pain by allied health professionals (the ALIGN trial): A cluster randomised trial protocol.** *Implem Sci* 2010, **5:**86.

24. Cobban SJ, Profetto-McGrath J: **Dental hygienists' research utilization: influence of context and attitudes.** *Int J Dent Hyg* 2011, **9:**191-198.

25. Najavits LM, Weiss RD, Shaw SR, Dierberger AE: *What do you think about treatment manuals questionnaire. Unpublished measure.* Belmont, MA: Harvard Medical School and McLean Hospital; 2000.

26. Najavits LM: *Protocol Implementation Scale. Unpublished measure.* Belmont, MA: Harvard Medical School and McLean Hospital; 1996.

27. Kim HS: *A study of three approahces to research utilization. Unpublished manuscript.* Kingston, Rhode Island: University of Rhode Island; 1988.

28. Walrath CM, Sheehan AK, Holden EW, Hernandez M, Blau MG: **Evidence-based treatments in the field: A brief report on provider knowledge, implementation, and practice.** *J Behav Health Serv Res* 2006, **33:**244-253.

29. Poissant L, Curran J: **The development of a questionnaire to assess organizational readniess to adopt e-health technologies.** Canadian Association for Health Services and Policy Research (CAHSPR) Conference; 2007.

30. Chandler GE: **The Relationship of Nursing Work.** *Unpublished doctoral dissertation* 1986, **University of Utah, UT.** .

31. Greenbaum P, Friedman RM, Kutash K: **Systems of care implementation survey.** *Research and Training Center for Children ’s Mental Health* 2008.

32. Chorpita et al cited in Lewis C C & Simons A D: **A pilot study disseminating cognitive behavioral therapy for depression: Therapist factors and perceptions of barriers to implementation.** *Adm Policy Ment Health* 2011, **38:**324-334.

33. Slee PT, Lawson MJ, Russell A, Askell-Williams H, Dix KL, Owens L, Skrypiec G, Spears B: **Kids Matter Primary Evaluation final report.** Centre for Analysis of Educational Futures, Flinders University of South Australia., 2009.

34. Thoresen CJ: **Antecedents and consequences of coping with setbacks at work: A theory drive framework.** Unpublished doctoral dissertation. University of Iowa, 2000.

35. Thompson CJ: **Extent and factors influencing research utilization among critical care nurses.** Unpublished doctoral dissertation. Texas Woman's University, 1997.

36. Doll WJ, Torkzadeh G: **The measurement of end-user computing satisfaction.** *MIS Quart* 1988, **12:**259-274.

37. Schultz RL, Slevin DP: **Implementation and organi-zational validity: An empirical investigation.** In *Implementing Operations Research/Management Science.* Edited by Schultz RL, Slevin DP. New York, NY: American Elsevier; 1975: 153-182

38. Davis FD: **Perceived usefulness, perceived ease of use, and user acceptance of information technology.** *MIS Quart* 1989, **13:**319-340.

39. Perciful EG: **The relationship between planned change and successful implementation of computer assisted instruction.** *Comput Nurs* 1992, **10:**85-90.

40. Jaana M, Pare G, Sicotte C: **Information technology capacities assessment tool in hospitals: instrument development and validation.** *Int J Technol Assess Health Care* 2009, **25:**97-106.

41. Kaya N: **Factors affecting nurses' attitudes toward computers in healthcare.** *Comput Inform Nurs* 2011, **29:**121-129.

42. Avolio BJ, Bass BM, Jung DI: **Re-examining the components of transformational and transactional leadership using the Multifactor Leadership Questionnaire.** *J Occup Organ Psychol* 1999, **72:**441-462.

43. Brett JLL: **Use of nursing practice research findings.** *Nurs Res* 1987, **36:**344-349.

44. Stacey D, Pomey M, O'Connor A, Graham I: **Adoption and sustainability of decision support for patients facing health decisions: an implementation case study in nursing.** *Implem Sci* 2006, **1:**17.

45. Soper B, Hanney S: **Lessons from the evaluation of the UK's NHS R&D Implementation Methods Programme.** *Implem Sci* 2007, **2:**7.

46. Kaariainen M, Kyngas H: **The quality of patient education evaluated by the health personnel.** *Scand J Caring Sci* 2010, **24:**548-556.

47. Gagnon M, Sanchez E, Pons J: **From recommendation to action: psychosocial factors influencing physician intention to use Health Technology Assessment (HTA) recommendations.** *Implem Sci* 2006, **1:**8.

48. Guilbert ER, Morin D, Guilbert AC, Gagnon H, Robitaille J, Richardson M: **Task-shifting in the delivery of hormonal contraceptive methods: validation of a questionnaire and preliminary results.** *Int J Nurs Pract* 2011, **17:**315-321.
